# Cerebrovascular events induced by venomous snake bites: A systematic review

**DOI:** 10.1016/j.heliyon.2025.e42779

**Published:** 2025-02-19

**Authors:** Jorge Vasconez-Gonzalez, Karen Delgado-Moreira, Juan S. Izquierdo-Condoy, María de Lourdes Noboa-Lasso, Esteban Gamez-Rivera, María Belén Lopez-Molina, Andrés López-Cortés, Andrea Tello-De-la-Torre, Alejandra Torres Cerda, Daniela Silva Martinod, Esteban Ortiz-Prado

**Affiliations:** a One Health Research Group, Faculty of Health Science, Universidad de Las Americas, Quito, Ecuador; b Program in Occupational Safety and Health, The University of Porto, Porto, Portugal; c Cancer Research Group (CRG), Faculty of Medicine, Universidad de Las Américas, Quito, Ecuador

**Keywords:** Snakebite envenomation, Neurovascular complications, Venom-induced stroke, Hemorrhagic stroke, Ischemic stroke, Neurological toxicity

## Abstract

Snake bites represent a critical public health issue, affecting approximately 2.7 million people globally each year. Around 20 % of snake species are venomous, and their venom contains a complex array of toxins that can cause multi-organ damage, particularly affecting the nervous system, leading to both ischemic and hemorrhagic cerebrovascular events. This systematic review aims to compile and analyze data on cerebrovascular events associated with venomous snakebites. A comprehensive literature search was conducted using Scopus, PubMed, SciELO, and LILACS databases, with search terms including (“snake bite” OR “viper bite”) AND (“stroke” OR “hemorrhagic stroke” OR “ischemic stroke”). Studies in English, Spanish, French and Portuguese were reviewed, yielding 52 eligible articles reporting 73 cases of stroke following snakebites. Most cases were attributed to snakes from the *Viperidae* family, with 67.12 % of cases occurring in males. Ischemic strokes were the most frequent, comprising 73.97 % of reported cases. The most affected systems were the nervous, cardiovascular, and respiratory systems. Snakes from the *Bothrops* genera and *Daboia russelii* specie caused the widest range of symptoms, including altered consciousness, ptosis, hypertension, drowsiness, aphasia, and tachycardia. Stroke is a severe complication of snakebite envenomation. Regarding treatment, the articles included emphasize the use of antivenom serum; however, they do not go into detail about the specific management of cutaneous stroke due to a snakebite, whether ischemic or hemorrhagic It is crucial to develop standardized protocols for the management of snakebite-induced strokes and to conduct further research to identify the snake species whose venom poses the highest risk for cerebrovascular complications.

## Highlights

• Ischemic stroke is the most common cerebrovascular event caused by snake venom.
• Snake venom induces strokes via thrombus formation, vasospasm, and vascular damage, leading to hemorrhage and coagulopathy.
• Daboia russelii and Bothrops species account for most snake venom-induced cerebrovascular events.

## Graphical abstract

**Figure:**
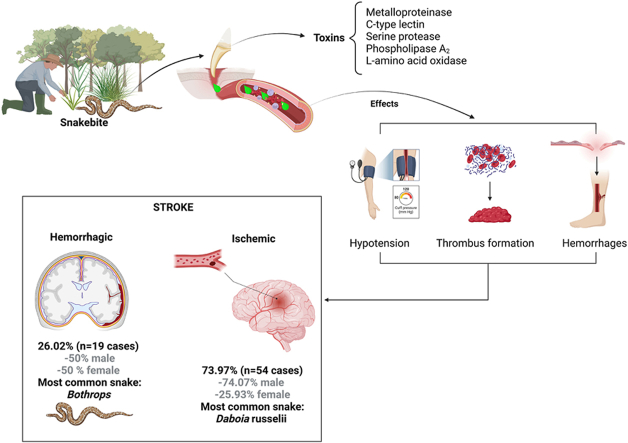

## 1 Introduction

Snakebites remain a significant public health issue today, particularly affecting workers and residents in rural areas or communities with lower socioeconomic conditions [1]. Due to its impact, snakebite envenomation has been classified by the World Health Organization (WHO) as a neglected tropical disease, affecting approximately 2.7 million people annually, with a mortality rate ranging from 81,000 to 138,000 deaths per year [2]. The highest mortality rates from snake bites are found in Asia (0.96/100,000) and Africa (0.44/100,000) [3]. In response to this critical issue, many studies have focused on assessing the burden of snakebites and the lethal, systemic, and local effects of snake venom toxins on the human body [[3], [4], [5]]. However, challenges remain, including the misidentification of snake species and limited access to healthcare for those affected in remote areas [[6], [7], [8]].

Due to climatic variations and biodiversity, countries with greater ecological diversity often have a higher number of snake species [9] Globally, out of the nearly 3,000 identified snake species, approximately 20 % are venomous and pose a significant threat to human health [10,11]. Some of the most important snakes worldwide include Russell's vipers, Malayan and lancehead pit vipers, rattlesnakes, puff adders, cobras, mambas, kraits, and taipans [12]. Snake venom is a complex mixture of enzymes, including phospholipase A2, acetylcholinesterase, hyaluronidase, and metalloproteinases, which exert neurotoxic, procoagulant, or fibrinolytic actions [13]. Following a bite, the venom is injected either subcutaneously or intramuscularly, depending on the fang size. Once in the body, the venom causes localized pathological effects on surrounding tissues, while some toxins spread systemically through the lymphatic system and bloodstream, enabling them to affect multiple organs [15].

Snakebite envenomation manifests with both local and systemic symptoms, primarily including local tissue damage, inflammation, microvascular injury causing hemorrhage, skeletal muscle necrosis with impaired muscle function, blister formation, and dermonecrosis [[16], [17], [18], [19]]. Coagulation abnormalities and hemolysis are also common, presenting as epistaxis, hematemesis, cutaneous ecchymosis, hemoptysis, subconjunctival hemorrhages, retroperitoneal bleeding, and gingival bleeding. Conversely, procoagulant effects can result in thrombotic microangiopathy [19]. Neurotoxic effects can result in ptosis, ophthalmoplegia, and paralysis of the intercostal muscles and diaphragm, ultimately leading to respiratory failure [19,20]. Direct myocardial damage can result in arrhythmias, bradycardia, tachycardia, and hypotension. Severe cases may progress to myocardial infarctions caused by factors such as hypovolemia, anaphylactic shock, coronary thrombosis, vasoconstriction, myocardial necrosis, and hemorrhage [19,21]. Snake envenomation can lead to acute kidney injury, with the potential to progress to chronic kidney disease or kidney failure. Additional common manifestations include nausea, vomiting, diaphoresis, syncope, drowsiness, dyspnea, hemiparesis, and hemiplegia [19,22].

The pathophysiological mechanisms of snake venom are multifaceted, driven by a range of toxins with distinct effects. Among the most significant are phospholipases A2, which induce local and systemic myotoxicity, pain, lymphatic vessel damage, edema, neurotoxicity, nephrotoxicity, and hemolysis; metalloproteinases, which contribute to myonecrosis, extracellular matrix degradation, blistering, pain, edema, cardiovascular shock, nephrotoxicity, and coagulopathy; hyaluronidases, which facilitate extracellular matrix degradation; three-finger toxins, which cause cytotoxicity, necrosis, and neurotoxicity; snake venom serine proteinases, which lead to coagulopathy, edema, and hypotension; disintegrins, which inhibit platelet aggregation; and natriuretic peptides, which result in hypotension [22].

Prompt treatment significantly improves the likelihood of full recovery and a rapid return to normal life [1]. For over 120 years, the cornerstone of snakebite treatment has been the use of animal-derived immunoglobulins (antivenoms) [1]. However, in many regions, there is a deficiency in both the production and accessibility of antivenoms, leading to variability in mortality rates [23]. In this context, the rarer yet severe complications, such as stroke, represent a significant public health challenge [24]. It is also important to recognize that severe complications, such as stroke, have a profound impact on patients' lives, especially in low-resource settings [25,26]. Stroke-related disabilities result in substantial health burdens, leading to long-term physical dependence, reduced productivity, and increased demand for healthcare services [[27], [28], [29]].

Therefore, it is crucial to analyze cerebrovascular complications induced by snake venom, as they represent an understudied health issue with potentially severe and long-lasting consequences for patients, especially those in rural areas with limited access to healthcare services. With this review, we aim to shed light on this little-known topic by compiling available information on cerebrovascular events related to venomous snakebites. By doing so, we hope to contribute to the development of more effective strategies for the management and prevention of these complications, ultimately improving clinical outcomes and reducing the burden of this neglected disease.

## 2 Methodology

### 2.1 Research questions

The central research questions guiding this systematic review are as follows:

• ***Can snake venom act as a risk factor for the development of hemorrhagic or ischemic strokes?***
• ***Which snake genera are most commonly associated with these complications?***

### 2.2 Study design

A systematic review of case reports and case series examining ischemic and hemorrhagic strokes following venomous snake bites was conducted. The review adhered to the PRISMA (Preferred Reporting Items for Systematic Reviews and Meta-Analyses) guidelines. The research protocol was registered in the PROSPERO database under the code: CRD42024583307. The full protocol is available at: https://www.crd.york.ac.uk/prospero/display_record.php?RecordID=583307.

### 2.3 Search strategies

The literature search was performed in both English, Spanish, Portuguese and French, with no restrictions on the publication date. The databases used for the search included PubMed, Scopus, SciELO, and LILACS. Additionally, the reference lists of the selected articles were manually reviewed to identify any studies that were not captured by the initial search. The following search syntax was used, incorporating indexed terms, keywords, and Boolean operators:

*(“snake bite” OR “viper bite”) AND (“stroke” OR “hemorrhagic stroke” OR “ischemic stroke").*

### 2.4 Selection criteria

#### 2.4.1 Inclusion criteria

• Studies involving human subjects.
• Studies where cerebrovascular events were confirmed by imaging tests such as CT or MRI.
• Case reports and case series that described stroke as a complication of snakebite.

#### 2.4.2 Exclusion criteria

• Studies where the term ‘stroke’ referred to cardiac output or stroke volume.
• Studies published in languages other than English or Spanish.
• Studies without imaging-confirmed cerebrovascular events.
• Animal studies.
• Studies focusing on neurological complications other than stroke.

### 2.5 Bias assessment

To minimize the risk of bias, data extraction was performed independently by three reviewers (JEV, KDM, and MLNL) at different times. Any discrepancies in the extracted data were resolved through discussion and consensus among the reviewers. The studies were critically appraised using the JBI (Joanna Briggs Institute) critical appraisal checklist for case reports and case series, ensuring that only moderate to high-quality studies were included in the final analysis.

### 2.6 Data synthesis

Following the selection process, a thorough review of all manuscripts that met the inclusion criteria was performed. Those that met the criteria underwent further review, during which they were assessed using the JBI critical appraisal tool for case reports and case series, ensuring rigor and reliability. All studies were evaluated and categorized based on their quality (Table S1, Table S2).

The main findings of the studies included in this systematic review were extracted and summarized in tables, which included information such as the person involved, the clinical presentation and results of the cerebrovascular event, treatment received, and the final outcome of each case.

By following a systematic approach, this review aimed to provide a detailed and reliable summary of the association between venomous snakebites and the risk of developing cerebrovascular complications, while also highlighting the need for further research in this area.

## 3 Results

**Fig. 1:**
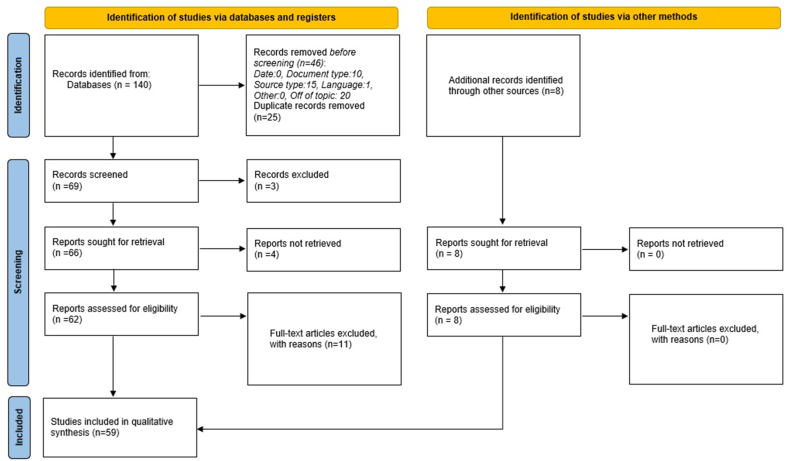
PRISMA flow chart of the study selection in this systematic review.

A total of 148 articles were initially identified, with 140 coming from database searches and 8 from citation searching. After removing 25 duplicates and 46 Records removed before (Off topic, Document type, Source type, Language), 69 records were screened, and 3 were excluded. Out of the remaining 66 records, 4 Only the abstract was accessed and not the full article. Ultimately, 62 full-text articles were assessed for eligibility, with 11 being excluded for various reasons. This left 51 articles, to which the 8 citation-searched records were added, bringing the total to 59 studies that met the inclusion criteria for this systematic review. Among these 59 studies, 4 were high-quality case series, and 55 were case reports, with 7 of the case reports being of moderate quality and 48 of high quality (see Fig. 1).

### 3.1 General description

#### 3.1.1 Gender distribution and stroke type in snakebite-induced cerebrovascular events

Cases were reported from 16 countries across Africa, Asia, Oceania, and the Americas, with the highest number of cases observed in India (27 cases), Brazil (13 cases), and Sri Lanka (12 cases). Among the total, 64.63 % (n = 53) of the cases occurred in males, while 32.92 % (n = 27) were in females. Additionally, the gender of two patient was not specified (Table 1). The mean age of the patients was 41.68 years (SD: 19.19), with men averaging 41.81 years (SD: 19.52) and women averaging 41.44 years (SD: 18.90).

**Table 1:** Summary of reported cases of ischemic and hemorrhagic strokes following snakebites. Cases span across 16 countries from Africa, Asia, Oceania, and the Americas. The table includes details on patient demographics, snake species involved, presenting symptoms, imaging findings, and stroke type, therapeutic interventions and outcome.

Author	Country	Sex	Age (years)	Snake	Snake Identification Method	Symptoms	Imaging Findings	Therapeutic interventions	Stroke Type	Outcome
Bashir & Jinkins 1985 [30]	Saudi Arabia	Female	13 years	*Echis carinatus*	Photograph	- Right hemiplegia - Aphasia	CT: Areas of low density in the left parietal region which enhanced irregularly following contrast administration with a serpiginous pattern	Five 10 ml intravenous injections of polyvalent anti-snake serum	Ischemic	Patient survives, has physical after-effects
Lee et al. 2001 [44]	Korea	Male	54 years	*Agkistrodon blomhoffii brevicaudus*	NA	- Dizzy - Nauseated - Vomit - Diplopia - Right hemiparesis - Hemifacial palsy	MRI: Acute infarction in left pontine region extending to basal surface and bilateral tegmentum of the pons	The patient was managed conservatively	Ischemic	Patient survives, has physical after-effects
Numeric et al 2002 [51]	Saint Lucian	Male	32 years	*Bothrops*	Description	- Left hemiplegia - Aphasia -Hypertension - Tachycardia - Fever	MRI: Multiple areas of cerebral ischemia, especially in the right anterior cerebral artery territory	30 mL of Wyeth polyvalent crotalidantivenin intravenously (IV), IV ceftazidime and IV met-ronidazole	Ischemic	Patient survives with minimal neurologic sequelae
Thomas et al 2006 [65]	Martinique	Male	46 years	*Bothrops*	Snake killed and captured	- Visual loss - Quadranopsia	MRI: Showed acute bilateral occipital infarcts	Three 20 ml intravenous injections of antivenom	Ischemic	Patient survives, and presents complete recovery of vision
Thomas et al 2006 [65]	Martinique	Male	55 years	*Bothrops*	NA	- Speech disorder - Right hemiparesis - Aphasia	MRI: Acute ischemic stroke in the left middle cerebral artery territory	Two 20 ml doses and one 40 ml dose of antivenom	Ischemic	Patient survives with neurological sequelae
Thomas et al 2006 [65]	Martinique	Male	66 years	*Bothrops*	Description	- Confusion - Agitation - Left hemiparesis - Left hemianopsia	MRI: Bilateral small hemispheric cortical infarcts	One 20 ml doses and one 40 ml dose of antivenom	Ischemic	Patient survives without significant disability
Santos-Soares et al. 2007 [80]	Brazil	Female	65 years	*Bothrops*	NA	- Motor deficit - Aphasia - Left deviation of labial commissure - Alteration of consciousness	CT: Hematoma in the left temporo-parietal lobe, with perilesional edema	The hematoma is evacuated directly through surgery.	Hemorrhagic	Patient survives and made a full recovery
Mugundhan et al. 2008 [48]	India	Male	14 years	NA	NA	- Ptosis - Altered sensorium - Anisocoria - Drowsy - Cellulitis on the right foot	CT: Bilateralcerebellar and right occipital infarct with mass effect.	Patient was treated with mannitol, frusemide, aspirin and antibiotics	Ischemic	Patient died
Narang et al 2009 [50]	India	Male	18 years	*Daboia russelii*	Snake killed and captured	- Speech disturbances - Right hemiplegia - Aphasia	MRI: Acute ischemic infarct in the left middle cerebral artery territory	A loading dose of 30 ml of polyvalent equine anti-snake venom, followed by continuous intravenous administration of 30 ml every 6 h by syringe pump until clotting time normalized, then a further dose of 30 ml was administered over 24 h, an injection of adsorbed tetanus toxoid was administered	Ischemic	Patient survives with good functional recovery
Gawarammana et al. 2009 [37]	Sri Lanka	Male	56 years	*Daboia russelii*	Snake killed and captured	- Ptosis - Ophthalmoplegia - Alteration of consciousness	CT: Acute multiple bilateral cerebral (both frontal and parietal lobes) and cerebella infarctions	20 vial of anti snake venom	Ischemic	Patient survives and made a full recovery
Gawarammana et al. 2009 [37]	Sri Lanka	Male	37 years	*Daboia russelii*	Snake killed and captured	- Ptosis - Ophthalmoplegia - Confusion - Hematuria - Left Hemiparesis - Alteration of consciousness	CT: Right-sided deep parietal (lentiform nucleus) infarction.	NA	Ischemic	Patient survives and made a full recovery
Gawarammana et al. 2009 [37]	Sri Lanka	Female	45 years	*Daboia russelii*	Snake killed and captured	- Ptosis - Ophthalmoplegia - Left hemiparesis - Confusion	CT: Acute infarction in the right frontal lobe and the right cerebellum	20 vials of anti snake venom	Ischemic	Patient survives and made a full recovery
Gawarammana et al. 2009 [37]	Sri Lanka	Female	45 years	*Daboia russelii*	Snake killed and captured	- Ptosis - Ophthalmoplegia - Seizures - Bleeding gums - Drowsy	CT: Acute ischemic infarctions of the head of the left caudate nucleus and right occipital lobe along with cerebral edema	20 vials of anti snake venom	Ischemic	Unable to walk, talk and attend to herself
Gawarammana et al. 2009 [37]	Sri Lanka	Male	28 years	*Daboia russelii*	Snake killed and captured	- Ptosis - Ophthalmoplegia - Seizures - Unconscious - Bleeding gums - Conjunctival hemorrhages - Hematuria	CT: infarctions of left parietal lobe and bilateral occipital lobes	20 vials of anti snake venom	Ischemic	Patient Died
Gawarammana et al. 2009 [37]	Sri Lanka	Male	53 years	*Daboia russelii*	Snake killed and captured	- Ptosis - Ophthalmoplegia - Acute renal failure	CT: Multiple bilateral cortical and cerebella ischemic infarctions	20 vials of anti snake venom	Ischemic	Residual motor weakness
Gawarammana et al. 2009 [37]	Sri Lanka	Male	35 years	*Daboia russelii*	Snake killed and captured	- Ptosis - Ophthalmoplegia - Alteration of consciousness - Dysphasia	CT: Ischemic infarction of the left frontal lobe	20 vials of anti snake venom	Ischemic	Expressive dysphasia
Gawarammana et al. 2009 [37]	Sri Lanka	Male	39 years	*Daboia russelii*	NA	- Ptosis - Ophthalmoplegia - Drowsy	CT: Multiple infarctions involving the cerebellum and occipital regions with peripheral edema	20 vials of anti snake venom	Ischemic	Residual motor weakness
Gawarammana et al. 2009 [37]	Sri Lanka	Male	54 years	*Daboia russelii*	NA	- Ptosis - Ophthalmoplegia - Left hemiparesis	CT: Acute parieto-temporal infarction	20 vials of anti snake venom	Ischemic	Patient survives and made a full recovery
Machado et al. 2010 [73]	Brazil	Female	62 years	*Bothrops*	NA	- Bleeding gums - Aphasia - Altered level of consciousness - Coluria - Hematuria - Asthenia Paresis of the right upper limb - Deviation of the lip commissure to the right - Hypertension	CT: Hemorrhagic lesions with perilesional edema of 20–30 mm in the left frontal and right parietal lobes and partially effaced cerebral sulci	Antivenous administration (dose not specified) and conservative treatment	Hemorrhagic	High blood pressure
Mechán Méndez et al. 2010 [74]	Peru	Male	20 years	*Bothrops*	NA	- Headache - Fever - Hyporexia - Right hemiparesis - Hematuria - Amaurosis - Alteration of consciousness - Gum bleeding - Aphasia - Dysarthria - Paresis of oculomotor muscles	CT: Intraparenchymal hemorrhagic stroke in left parieto-occipital area	25 mg of Dexamethasone antivenom (24 mg, for one day only), Mannitol, Ranitidine and analgesics	Hemorrhagic	Complete loss of vision in the right eye and partial loss of vision in the left eye
Chani et al. 2010 [82]	Morocco	NA	55 yeas	*Cerastes cerastes*	NA	- Confused - Agitated - Hypotension - Tachycardia	CT: ischemic lesions, frontal and parieto-occipital, with hemorrhagic softening	Antivenous serum perfusion in six ampoules on the first day, after six ampoules were administered, associated with vascular filling with crystalloids, probable antibiotic therapy based on amoxicillin-clavulanic acid, analgesia with morphine, and local care in the bite area, in addition to calcium heparin in prophylactic doses.	Ischemic	No sequelae
Gouda et al. 2011 [40]	India	Female	40 years	*Daboia russelii*	Description	- Altered sensorium - Drowsy - Hypotonia - Cellulitis on left foot	MRI: Hypo-intensities in both lobes of cerebellum, vermis, cerebellar tonsils, and both occipital lobes	24 vials of anti snake venom	Ischemic	Significant ataxia of gait,
Deepu et al. 2011 [36]	India	Female	48 years	*Daboia russelii*	Snake killed and captured	- Altered sensory function - Imminent respiratory arrest	CT: Infarction affecting the right cerebellar hemisphere, the medulla oblongata and the pons with mass effect and obstructive hydrocephalus	Decompressive craniectomy	Ischemic	Glasgow scale 2/15
Ittyachen & Mohan 2012 [41]	India	Male	55 years	*Daboia russelii*	Snake killed and captured	- Drowsy - Disorientation	MRI: Bilateral infarcts of the thalamus.	24 vials of anti snake venom and tetanus toxoid (doses not specified)	Ischemic	Patient survives and made a full recovery
Bhatt et al 2013 [32]	India	Male	65 years	NA	NA	- Unconscious - Bleeding from the mouth - Slurring of speech - Right hemiparesis - Cellulitis of the left lower limb - Hypotension	MRI: Acute ischemic infarction on the left side in the precentral and postcentral gyrus, hemipons and cerebellum	Anti snake venom, ceftriaxone and clindamycin alongside supportive therapy (doses not specified), four units of fresh frozen plasma	Ischemic	Patient survives and made a full recovery
Vale et al. 2013 [66]	Brazil	Male	16 years	*Crotalus durissus terrificus*	Description	- Drowsy - Palpebral weakness - Diplopia - Headache - Visual impairment	MRI: Left temporo-occipital infarct with areas of ischemia in the pons and cerebellum	20 vials of anti snake venom	Ischemic	Severe behavioral and cognitive impairment
Aissaoui et al. 2013 [103]	Morocco	Male	73 years	*Cerastes cerastes*	Description	- Precordialgia - Nausea - Vomiting - Hypertension - Dysarthria - Left homonymous hemianopia.	CT: presence of a left occipital ischemic lesion and left temporoparietal petechial hemorrhagic lesions	Paracetamol and nefopam, anti-tetanus prophylaxis	Ischemic	Patient survived
Subasinghe et al 2014 [63]	Sri Lanka	Female	54 years	*Daboia russelii*	Sanke captured	- Hematuria - Loss of vision	CT: Bilateral posterior circulation infarcts without hemorrhages	10 vials of anti snake venom	Ischemic	Mild improvement of visual acuity
Mahale et al. 2014 [45]	India	Male	58 years	*Trimeresurus gramineus*	Description	- Diminution of vision - Hypertension - Tachypnea - Cellulitis on the right foot	MRI: Hyperintensity in bilateral occipital lobe	3 vials of anti snake venom antibiotics, anti-platelets, nimodipine (60 mg every 6th hour), and anti-edema agents	Ischemic	Patient survives
Bush et al. 2014 [34]	USA	Male	50 years	*Crotalus oreganus helleri*	Description	- Hypertension - Tachycardia - Respiratory difficulties - Paresthesia - Difficulty speaking - Right hemiparesis - Right facial paresis	MRI: Acute infarcts affecting the right frontal lobe and the left parietal and occipital lobes CT: Extensive bilateral ischemic infarcts and cerebral edema,	12 vials of Crotalidae polyvalent immune Fab	Ischemic	Patient died
Bush et al. 2014 [34]	USA	Male	17 years	*Crotalus oreganus helleri*	Sanke captured	- Paresthesia in the right upper extremity - Facial paresis - Weaknesses of the upper and lower extremities	MRI: Acute infarction in the right sylvian and parasylvian region together with small, scattered foci of acute infarction seen in the bilateral frontal and occipital lobes, as well as in the right cerebellar hemisphere	26 vials of FabAV	Ischemic	Alteration in gait and mobility of the left arm
Gopalan et al. 2014 [39]	India	Female	32 years	*Daboia russelii*	NA	- Altered sensorium - Aphasia - Drowsy - Right hemiplegia	CT: Large ischemic infarct left side with midline shift.	20 vials of anti snake venom, intravenous antibiotics, mannitol and dexamethasone, (aspirin 150 mg/day	Ischemic	Walking with support, unable to speak, made incomprehensible sounds
Paul et al. 2014 [52]	India	Male	36 years	*NA*	NA	- Seizures - Alteration of consciousness - Anisocoria - Decreased pupillary reflex. - Left horizontal gaze palsy	MRI: Acute infarcts in left cerebellar hemisphere, bilateral occipital lobe and left thalamus	Anti snake venom, tetanus toxoid, antibiotics, anti-edema measures and anti-platelets	Ischemic	Patient survives
Paul et al. 2014 [52]	India	Male	40 years	*NA*	NA	- Drowsy - Aphasia	MRI: Infarct in the superior division of left middle cerebral artery	Anti snake venom and supportive treatment	Ischemic	Residual dysfunction of naming.
Rebahi et al. 2014 [57]	Morocco	Female	32 years	*Cerastes cerastes*	Description	- Seizures - Alteration of consciousness - Bleeding gums	CT: Extensive infarction of the brain including frontal, temporal, and parietal lobes with cerebral edema	Fasciotomy and supportive management	Ischemic	Patient died
Rebahi et al. 2014 [57]	Morocco	Female	5 years	*Cerastes cerastes*	Description	- Confusion - Tachypnea - Alteration of consciousness	CT: Right extensive frontal temporo-parieto-occipital ischemic stroke	Fasciotomy	Ischemic	Patient died
Rebahi et al. 2014 [57]	Morocco	Male	51 years	*Cerastes cerastes*	Description	- Tachycardia - Hypertension - Alteration of consciousness	CT: Acute bilateral cerebral infarctions in internal capsules	Supportive therapy, fresh frozen plasma and units of platelets were administered and also vitamin K, fasciotomy	Ischemic	Patient survive with normal neurologic examination
Kumar et al 2015 [42]	India	Male	32 years	*Daboia russelii*	NA	- Altered sensorium - Irritable - Restless - Bleeding from the mouth - Tachycardia - Tachypnea	MRI: Extensive infarctions involving right temporal, parietal, occipital lobes and small infarct in the left cerebellar hemisphere	10 vials of anti snake venom followed by seven vials three times a day for a period of five days; fresh frozen plasma, injection tetanus toxoid and prophylactic antibiotics	Ischemic	Weakness of all the four limbs
Pardal et al. 2015 [76]	Brazil	Male	10 years	*Bothrops marajoensis*	NA	- Drowsy - Hematuria - Comatose state - Right hemiplegia - Labial commissure deviation to the left	CT: Intracerebral hemorrhage in the right frontal area	12 vials of anti-snake venom	Hemorrhagic	Right flaccid hemiplegia
Ghezala & Snouda 2015 [104]	Tunez	Male	37 years	*Cerastes cerastes*	Description	- Vomiting - Hypotension - Altered state of consciousness - Left anisocoria - Cerebral rigidity	CT: Subarachnoid hemorrhage with areas of ischemia, subfactorial involvement, subdural hematoma and intraventricular hemorrhage	6 ampoules of antivenom, associated with volume expansion with crystalloids, antibiotic therapy with amoxicillin-clavulanic acid, analgesia with paracetamol	Hemorrhagic	Patient died
Prabhakar et al. 2016 [78]	India	Male	44 years	*Daboia russelii*	NA	- Diplopia - Ptosis - Ophthalmoparesis - Drowsy	CT: symmetrical hypodensity involving the subcortical white matter and centrum semiovale with a focus of hemorrhage in the posterior part of the right centrum semiovale MRI: Bilateral and symmetrical periventricular white matter hyperintensity which is also involving the subcortical white matter.	Anti snake venom (doses not specified)	Hemorrhagic	Recovered completely with no residual deficits
Cañas 2016 [35]	Colombia	Female	48 years	*Bothrops atrox*	Analysis conducted by biologist from a photograph	- Bleeding gums - Hematomas in both arms - Hemorrhagic blisters on the right leg - Hypotonia - Miotic pupils - Absent pupillary reflex	MRI: ischemia in multiple vascular territories, the greatest of them involving to brainstem. There was an image suggestive of a thrombus in the basilar artery	9 vials of anti snake venom	Ischemic	Patient was transferred for palliative care
Bhojaraja et al. 2016 [33]	India	Female	45 years	NA	NA	- Unconscious - Bleeding from the mouth - Hypotension	MRI: Acute to sub-acute infarcts in bilateral cerebellar hemispheres, bilateral thalami, bilateral frontal and parietal lobes, right centrum semi-ovale, right temporal lobe and right half of midbrain with hemorrhagic transformation	20 vials of anti snake venom, intravenous mannitol	Ischemic	Decreased left upper and lower meborium
Silveira et etl 2016 [81]	Brazil	Male	52 years	*Bothrops* spp	Snake killed and captured	- Vomiting - Altered behavior - Disorientation - Aggressiveness - Drowsy	CT: Hemorrhagic stroke in the left hemisphere	600 mg, of anti snake venom	Hemorrhagic	NA
Delgado et al. 2017 [71]	Brazil	Female	58 years	*Bothrops* spp	NA	- Deterioration of consciousness - Speech disturbance - Left hemiparesis	CT: nucleocapsular hematoma, flooding of the right ventricular system and appearance of hemorrhagic component in the subarachnoid region of the frontal and parietal lobes.	Anti snake venom (9 ampoules of 10 ml)	Hemorrhagic	Improvement of motor and speech domains
Pothukuchi et al. 2017 [55]	India	Male	70 years	*Daboia russelii*	Snake killed and captured	- Bleeding gums - ptosis - Seizures	CT: Acute infarcts in left capsuloganglionic area	20 vials of anti snake venom along with repeated doses of Neostigmine 0.5 mg IM atropine 0.6 mg IV, Tetanus toxoid, levetiracetam 500 mg tablet orally twice daily, clopidogrel 75 mg per day orally.	Ischemic	Patient survived
Pothukuchi et al. [55]	India	Male	55 years	*Daboia russelii*	Snake killed and captured	- Ptosis	CT: Acute ischemic infarcts in bilateral frontal lobes	30 ml loading doce of anti snake venom, followed by continuous intravenous administration of 30 ml of anti snake venom in normal saline, neostigmine 0.5 mg IM atropine 0.6 mg IV, tetanus toxoid, ceftriaxone	Ischemic	Patient survived
Rathnayaka & Ranathunga 2017 [56]	Sri Lanka	Male	53 years	*Daboia russelii*	NA	- Ptosis - Bleeding gums - Hypertension - Tachypnea - Respiratory failure - Left hemiparesis	CT: Bilateral cerebral infarcts only on parietal lobes	20 vial of anti snake venom, hemodialysis, clopidogrel 75 mg	Ischemic	Patient died
Pothukuchi et al. 2018 [54]	India	Male	55 years	*Daboia russelii*	Sanke captured	- Ptosis - Left hemiparesis - Speech disturbances.	CT: Infarcts in bilateral frontal lobes	3 vials of anti snake venom, followed by continuous intravenous administration of another 3 vials of anti-snake venom in normal saline. When appears 10 vials was given as infusion followed by again 10 vials of as infusion after 1 h along with atropine and neostigmine. tetanus toxoid and antibiotics, aspirin and clopidogrel	Ischemic	Decreased strength and difficulty speaking
Sahoo & Sriramka 2018 [58]	India	Male	36 years	*NA*	NA	- Tachycardia - Alteration of consciousness	CT: Acute ischemic infarct in the left middle cerebral artery territory with edema	10 vials of anti snake venom, follow by continuous intravenous administration of 50 ml every 6 h by infusion	Ischemic	Patient needs to walk with support
Sahoo et la. 2018 [59]	India	Male	36 years	*Daboia russelli*	NA	- Alterations of consciousness - Hematemesis - Cellulitis on the right foot - Anisocoria - Decreased pupillary reflex	CT: Multiple infarctions involving left frontotemporal lobe, right basal ganglia, right thalamus, occipital lobe, and cerebellar region	30 vial of anti snake venom and mannitol injection	Ischemic	Residual weakness of power 3/5 in the right upper and lower limb
Zhang et al 2017 [68]	China	Male	34 years	*Deinagkistrodon acutus*	NA	- Tachycardia - Hypotension - Anisocoria	CT: Cerebral infarction and hernia in right brain	Anti snake venom, fasciotomy, he was transfused with 400 ml of blood, red blood cell for 2U	Ischemic	Patient died
Zeng et al. 2019 [67]	China	Female	49 years	*Trimeresurus stejnegeri*	Snake killed and captured	- Speech disturbances - Right hemiparesis - Aphasia - Deviation of the tongue to the right	MRI: Acute ischemic infarct in the left territory	Injection of polyvalent anti-snake venom serum, neuroprotective therapy, and anti-platelet aggregate treatment.	Ischemic	Mixed aphasia
Lahiri et al. 2019 [43]	India	Male	50 years	NA	NA	- Seizures - Aphasia - Right hemiparesis	CT: Multiple areas of hypodensity in the bilateral middle cerebral artery territories, involving caudate nucleus and parietotemporal cortex on the left side and frontoparietal cortex on the right side MRI: Acute malignant infarction in the left middle cerebral artery territory along with small infarct in the right middle cerebral artery territory	Intravenous phenytoin, 20 vials of anti snake venom	Ischemic	Broca's aphasia; , motor deficit on the right side
Pérez-Gómez et al. 2019 [77]	Brazil	Male	15 years	*Bothrops atrox*	NA	- Unconscious - Seizures - Anisocoria - Absence of pupillary reflex	CT: Intraparenchymal hemorrhage in the right frontal region with perilesional edema	12 ampoules of botropic antivenom	Hemorrhagic	NA
Pérez-Gómez et al. 2019 [77]	Brazil	Female	78 years	*Bothrops atrox*	NA	- Alteration of consciousness - Left hemiparesis - Seizures	CT: Hypodense lesion in the right frontal lobe, cortical-subcortical, and perilesional edema	12 ampoules of botropic antivenom	Hemorrhagic	Patient survived
Pérez-Gómez et al. 2019 [77]	Brazil	Female	20 years	*Bothrops atrox*	NA	- Headache - Hematemesis - Syncope - Hypotension - Seizures - Conjunctival hemorrhage - eyelid ecchymosis - Left hypoesthesia	CT: Hyperdensity in the posterior horn of the left lateral ventricle, with dilation of the homolateral temporal horn, related to the hemoventricle.	10 ampoules of botropic antivenom, volemic expansion, hydrocortisone, and promethazine, Amoxicillin-clavulanic acid	Hemorrhagic	Patient survived
Smith and Brown et al. 2019 [61]	Australia	Male	2 years	*Pseudonaja textilis*	Specialized testing of his blood	- Alteration of consciousness - Dysphagia - Respiratory distress.	MRI: Multiple supra and infratentorial anterior and posterior circulation infarcts involving the white matter, deep gray matter, nuclei, and cerebellar hemispheres	Aciclovir, ceftriaxone, methylprednisolone, the de patient The patient was managed conservatively with supportive treatment. Antibiotics, antivirals, and corticosteroids were ceased.	Ischemic	Near normal function.
Sachett et al 2020 [79]	Brazil	Female	65 years	*Bothrops atrox*	Description	- Dizziness - Headache - Vomit - Hypertension - Bradycardia - Tachypnea - Alteration of consciousness	CT: Subarachnoid hemorrhage with hydrocephalus and hemoventricle	IV promethazine, IV dipyrone, and IV hydrocortizone, 12 vials of Bothrops-Lachesis antivenom, mannitol, metoclopramide, midazolam, and furosemide	Hemorrhagic	Patient survived
Sachett et al 2020 [79]	Brazil	Male	22 years	*Bothrops atrox*	Description	- Headache - Tachycardia - Eye pain - Aphasia - Alteration of consciousness - Hypertension - Tachycardia	CT: Right frontoparietal hemorrhage, intraparenchymal hemorrhage already open to the ventricle, edema that deviated from the midline, and subarachnoid hemorrhage	12 vials of Bothrops-Lachesis antivenom	Hemorrhagic	Patient died
Dabilgou et al. 2021 [70]	Burkina Faso	Female	55 years	NA	NA	- Bleeding gums - Headache - Vomiting - Fever - Left hemiplegia - Tachycardia	CT: Heterogeneous hyperdensity in the internal capsule, lenticular nuclei in the right hemisphere and cerebral edema	Anti-snake venom and anti-tetanus serum, ceftriaxone 2 g/24 h, metronidazole 500 mg three time per day.	Hemorrhagic	Incomplete motor recovery
Dabilgou et al. 2021 [70]	Burkina Faso	Male	16 years	NA	NA	- Headache - Loss of consciousness - Ulcer on left arm - Hypertension - Polypnea	CT: Bilateral spontaneous left cerebellar and frontal hemorrhage,	Blood transfusion of 2 blood bags, ceftriaxone and received a dose of antivenom serum	Hemorrhagic	Patient recovered completely
Dabilgou et al. 2021 [70]	Burkina Faso	Female	30 years	NA	NA	- Motor deficit - Language disorder - Conscious disorder - Hypertension - Fever - Hematuria - Aphasia - Left ptosis	CT: Spontaneous temporary hematoma, perihematomal edema and left parietal dural hematoma	Antivenom serum, anti-tetanic vaccine, vitamin K, diazepam, and paracetamol, blood transfusion of 2 red globular pellet pockets	Hemorrhagic	Motor deficit
Yalcouyé et al 2021 [105]	Mali	Male	6 years	NA	NA	- Bleeding gums - Abdominal pain - Headache - Mydriasis - Ptosis - Blindness	CT: left frontal intraparenchymal hematoma extending to the region crossed by the optic pathways	Intravenous paracetamol (60 mg/kg/day), 20 % mannitol at 5 ml/kg/day in two doses a day and three doses of multivalent antivenom	Hemorrhagic	Blindness
Sirur et al. 2022 [60]	India	Female	60 years	*Hypnale*	Sanke captured	- Alteration of consciousness - Anuria - Vomit	CT: Large pontine bleed	38 vials of antivenom serum in 4 separate infusions, antihistamines and adrenaline	Hemorrhagic	On telephonic follow-up, no clinical symptoms of renal dysfunction or bleeding diathesis were reported
Sirur et al. 2022 [60]	India	Male	25 years	*Hypnale*	Photograph	- Alteration of consciousness - Right hemiparesis	CT: Middle cerebral artery territory infarct with midline shift	30 vials of antivenom serum	Ischemic	Patient died
Ansoumane Hawa et al. 2022 [69]	Morocco	NA	56 years	NA	NA	- Right hemiplegia - Right anisocoria - Gangrene on the right foot - Hypotension	CT: Subarachnoid meningeal hemorrhage and intraparenchymal hematoma	IV infusion of antivenom at a dose of four ampoules, along with crystalloid volume expansion, intravenous antibiotic therapy, and local care of the bite site	Hemorrhagic	Patient died
Martinez-Villota et al. 2022 [46]	Colombia	Male	50 years	*Bothrops*	NA	- Left hemiparesis - Alteration of consciousness	MRI: Multiple areas of bilateral fronto-temporal, and occipital ischemia	9 vials of antivenom serum	Ischemic	Bilateral paresis on the VI and III cranial of the left side
Namal Rathnayaka et al. 2022 [49]	Sri Lanka	Male	71 years	*Hypnale* spp	NA	- Deviation of the mouth to the right - Reduced muscle power - Slurring of speech - Hypertension	CT: Ischemic infarct in right-sided internal capsule	Aspirin 300 mg, clopidogrel 75 mg, and atorvastatin 20 mg, Intravenous clindamycin 600 mg 6-hourly and cefotaxime 1 g 8-hourly	Ischemic	Limb weakness
Pinzon et al. 2022 [53]	Indonesia	Male	72 years	*Calloselasma rhodostoma*	NA	- Hypertension - Tachypnea - Left hemiparesis - Headache	CT: Ischemic infarct in the pericallosal of the right lateral periventricular anterior horn	Two vials of antivenom serum in 500 mL of sodium chloride, travenous ketorolac 10 mg and ceftriaxone 1 g twice a day	Ischemic	Patient survived
Ghosh et al. 2022 [38]	India	Female	40 years	*Daboia russelii*	Photograph	- Tachycardia - Headache - Vomiting - Blurred vision - Papilledema - Hematuria	MRI: Signal hyperintensity on T1, T2,	10 vials of anti-snake venom, ere diluted in 400 ml normal saline and administered over 60 min, IV pantoprazole 40 mg/day oral cefuroxime axetil 1000 mg/day, 0.5 mL intramuscular tetanus toxoid, 20 extra vials	Ischemic	Patient survived
Ouedraogo et al. 2022 [106]	Burkina Faso	Male	60 years	*NA*	NA	- Tachycardia - Fever - Edema in the right lower limb - Altered state of consciousness	CT: showed multiple intracranial bleeding at the occipital, cerebellar and ventricular levels	Ceftriaxone 2 g, metronidazole 500 mg, multipurpose anti-venom Afrique (2 ampoules), anti-tetanus vaccine and serum, paracetamol 1 g/8 h, nefopam 20 mg/8 h, amoxicillin and clavulanic acid 1.2 g/8 h, metronidazole 500 mg/8 h and tranexamic acid 1 g as a bolus and then 1 g as an infusion over 8 h.	Hemorrhagic	Sequelae of tetraparesis
Assamadi et al. 2022 [107]	Morocco	Female	6 years	NA	NA	- Confused - Conjunctival discoloration - Tachycardia - Hypotension - Left hemiparesis - Hematoma in the left lower limb - Edema in the left lower limb - Hyperreactive pupils	Brain CT revealed a focus of right temporoparietal ischemia systematized with subarachnoid hemorrhage and mass effect on the ipsilateral lateral ventricle and the onset of subfalcorial involvement.	Antivenom serum	Ischemic	Patient died
Senthilkumaran et al. 2023 [83]	India	Male	40 years	*Daboia russelii*	Identified by a herpetologist	- Bleeding in the gums - Localized swelling - Right axillary lymphadenopathy -Palpitations - Nausea - Abdominal pain - Hypotension - Tachycardia - Restless - Irritable	Axial non-contrast CT scan of the brain corroborating acute hemorrhage of the pituitary gland	5 vials of polyvalent antivenom, two doses of intramuscular epinephrine and dexamethasone, 10 extra vials of polyvalent antivenom intravenously over 1 h, noradrenaline, hydrocortisone 50 mg intravenously twice daily for five days and thyroxine 150 μg	Hemorrhagic	Patient survived
Bentes et al. 2024 [31]	Brazil	Female	54 years	*Bothrops atrox*	NA	- Hypotension - Epistaxis - Bleeding from oral mucosa - Altered consciousness - Seizures - Tachycardia	CT: Left hemispheric hypodensity, effacement of sulci, and 10 mm - midline shift	12 vials of *Bothrops* antivenom, 0.05 mg/mL of fentanyl and 5 mg/mL of midazolam, fresh frozen plasma (one unit) packed red blood cells (two units)	Ischemic	Aphasia, but with spontaneous eye opening, motor response only occurring with painful stimulus, and right hemiplegia.
Srinath et al. 2024 [62]	India	Male	50 years	*Daboia russelii*	Photograph	- Dysphonia - Drowsy - Dysarthria - Ptosis - Slurring of speech - Giddiness - Vomit - Dysdiadochokinesia	MRI: Bilateral cerebellar infarct with the left superior cerebellar peduncle showing restricted diffusion and low apparent diffusion coefficient values	10 vials of antisnake venom, neostigmine and atropine injections, antiplatelet therapy, neuroprotective medications, high-dose statins	Ischemic	Upper-limb incoordination and mild gait ataxia
Sun et al. 2024 [64]	Taiwan	Male	88 years	*Protobothrops mucrosquamatus*	Photograph	- Hypertension - Left hemiplegia - Altered consciousness	MRI: acute occlusion in the right middle cerebral arterial M2 region and acute ischemic injury extending from the right frontotemporal lobes to the right insula	1 vial of freeze-dried hemorrhagic antivenom, then received an additional six vials, aspirin 300 mg and clopidogrel 300 mg	Ischemic	NA
Nascimento et al. 2024 [75]	Brazil	Male	52 years	*Bothrops atrox*	Identified by a herpetologist	- Bleeding gums - Hematemesis - Cardiorespiratory arrest - Comatose - Acute kidney failure	CT: Angioedema of the bilateral occipital lobe, white and gray matter with an abnormal attenuation coefficient, compatible with hemorrhagic stroke	6 vials of Bothrops antivenom, 2 hemodialysis sessions	Hemorrhagic	Patient died
Ladgani et al. 2024 [72]	Morocco	Male	15 years	NA	NA	- Altered consciousness - Hypotension	CT: Meningeal hemorrhage, intracerebral hematomas and diffuse ischemic lesions	Antivenom,	Hemorrhagic	Patient died
Senthilkumaran et al. 2024 [83]	India	Female	22 years	*Bungarus caeruleus*	Identified by a herpetologist	- Bilateral ptosis - Difficulty breathing - Headache - Nausea	Multimodal magnetic resonance angiography revealed multifocal segmental cerebral artery vasoconstriction, predominantly affecting both the middle cerebral arteries and the P1-segment of the posterior cerebral artery	Intravenous infusion of 10 vials of polyvalent antivenom, oral nimodipine, ravenous infusion of paracetamol and ibuprofen	Ischemic	No permanent or prolonged neurological deficits
Senthilkumaran et al. 2024 [84]	India	Female	25 years	*Daboia russelii*	Identified by a herpetologist	- Headache	Multimodal magnetic resonance angiography segmental vasoconstriction in the bilateral middle and posterior cerebral arteries with no systemic bleeding.	25 vials of polyvalent antivenom, Multimodal magnetic resonance angiography nimodipine	Ischemic	No neurological deficit
Senthilkumaran et al. 2024 [84]	India	Female	25 years	Cobra	Identified by a herpetologist	- Breathlessness - Hypertension - Tachycardia - Bilateral ptosis - Restricted eye movement	Multimodal magnetic resonance angiography segmental revealed segmental areas of luminal narrowing with skip areas, giving a beaded appearance in proximal segments of the bilateral anterior, middle and posterior cerebral arteries	IV atropine (2 mg), neostigmine (0.5 mg), 20 vials of polyvalent antivenom	Ischemic	No sequelae

CT: Computed tomography, MRI: Magnetic resonance imaging.

The most frequent type of cerebrovascular event was ischemic, occurring in 71.95 % (n = 59) of cases [[30], [31], [32], [33], [34], [35], [36], [37], [38], [39], [40], [41], [42], [43], [44], [45], [46], [47], [48], [49], [50], [51], [52], [53], [54], [55], [56], [57], [58], [59], [60], [61], [62], [63], [64], [65], [66], [67], [68]], while hemorrhagic stroke occurred in 28.04 % (n = 23) of cases [60,[69], [70], [71], [72], [73], [74], [75], [76], [77], [78], [79], [80], [81]]. When differentiating by gender, 67.79 % (n = 40) of ischemic strokes occurred in the male population [32,34,34,37,41,[43], [44], [45], [46], [47], [48], [49],[51], [52], [53], [54], [55], [56], [57], [58], [59], [60], [61], [62],[64], [65], [66],^68^], and 56.52 % (n = 13) of hemorrhagic stroke occurred in the male population while female population represents the 39.13 % (n = 9) of the cases of hemorrhagic stroke (31,41–52), 1 case of hemorrhagic stroke and 1 case of ischemic stroke are not described whether it occurred in men or women [69,82].

### 3.2 Identification of snake genus in stroke-related envenomation cases

In 81.60 % (n = 67) of the cases, the genus of the snake responsible for the bite of the patients was identified [30,^31^,[39], [38], [37], [36], [35], [34], [42], [41], [40],[44], [45], [46], [47],[49], [50], [51],[53], [54], [55], [56], [57], [58], [59], [60], [61], [62], [63], [64], [65], [66], [67], [68],^71^,[73], [74], [75], [76], [77], [78], [79], [80], [81], however, in 15 cases the genus of the snake could not be identified [32,33,43,48,52,58,69,70,72]; the majority of the cases 37.31 % (n = 25) were caused by snakes of the genus *Daboia russelii* followed by the genus *Bothrops* 28.35 % (n = 19) (Table 2). It is important to highlight that of the 70 cases in which the responsible snake was identified, only 6 cases specify that the identification was carried out by a specialist; 1 case was identified by a biologist and 5 cases by a herpetologist [35,75,83,84].

**Table 2:** Distribution of stroke cases by snake genus, geographic region, stroke type, and common symptoms associated with envenomation. The table illustrates the frequency and distribution of cerebrovascular events caused by different snake genera across multiple regions, detailing the type of stroke (ischemic or hemorrhagic) and the most frequently reported symptoms. It also contains the proteomic analysis of the venom of each of the snakes.

Snake Genus	n	%	Geographic Distribution	Stroke Type	Most Common Symptoms	Proteomic Analyses
*Agkistrodon blomhoffii brevicaudus*	1	1.49	Korea	Ischemic	Dizziness, hemiparesis, diplopia	L-amino acid oxidase, metalloproteinase, glutaminyl cyclase, salmobin, plasminogen activator, halystase, phospholipase A2 [108]
*Bothrops∗∗*	19	28.35	Brazil, Peru, Colombia, Martinique	Ischemic, Hemorrhagic	Hemiplegia, aphasia, visual impairment	Metalloenzyme, Batroxhragin, Atroxlysin III, HI-5, Batx-I, Atroxlysin I, Atroxlysin Ia, Batroxase, Batroxostatin, Batroxobin, BA III-4, SVSPs, Thrombocytin, BaPLA2-I, BaPLA2-III, *B. atrox* PLA2, Mytoxin I, BaTX-I, BaTX-II, Ba PLA2, Galatrox, *B. atrox* LAAO, *B. atrox* LAAO, Hyal-Ba, BPP-BAX12, Nucleotidase, Phosphodiesterases [109]
*Calloselasma rhodostoma*	1	1.49	Indonesia	Ischemic	Hemiparesis, hypertension, headache	Metalloproteinases, which the P-I and P-II classes predominate, C-type lectins, snake venom serine protease, L-amino acid oxidase, phospholipase A2, cysteine-rich secretory protein nerve growth factor, neurotrophin, phospholipase B, 5′ nucleotidase and phosphodiesterase, totaling [96]
*Cerastes cerastes*	6	8.95	Morocco	Ischemic	Seizures, hemiplegia, alteration of consciousness	Disintegrins, procoagulant snake venom serine proteinase, cerastocytin, phospholipase A_2_, C-type lectin-like proteins and metalloproteinases [110]
*Crotalus*∗∗	3	4.47	USA, Brazil	Ischemic	Hemiparesis, facial paresis, hypertension	CTL, Dis, CRiSP, SVSP, SVMP-P I-III, and LAAO [111]
*Daboia russelii*	25	37.31	India, Sri Lanka, Bangladesh	Ischemic, Hemorrhagic	Ptosis, hemiparesis, ophthalmoplegia	A Chain A, Phospholipase A2 VRV-PL-VIIIa, Acidic phospholipase A2 daboiatoxin A, Basic phospholipase A2 3, Basic phospholipase A2 Drk-b1, Basic phospholipase A2 Drk-b2, Basic phospholipase A2 RVV-VD, Daboxin P, Phospholipase A2-II, Factor V activator RVV-V alpha, A Chain A, Vipera russelli proteinase RVV-V gamma, Cysteine-rich seceretory protein Dr-CRPK, Cysteine-rich seceretory protein Dr-CRPB, Cysteine-rich seceretory protein, Zinc metalloproteinase-disintegrin-like daborhagin-K, Kunitz-type protease inhibitor, Vascular endothelial growth factor A isoform 1, VR-1 precursor, Secreted L-amino acid oxidase precursor, Beta-nerve growth factor, Glutaminyl-peptide cyclotransferases [112]
*Deinagkistrodon acutus*	1	1.49	China	Ischemic	Hemiplegia, anisocoria, hypotension	Phospholipase A2, Snaclec agkicetin-C subunit alpha, Antithrombin A B-chain, Lys-49 phospholipase A2 precursor, Thrombin-like protein acutobin precursor, Basic phospholipase A2 DAV-N6, Antithrombin 1 B chain, Snake venom metalloproteinase aculysin-1, Snaclec anticoagulant protein subunit A, Snaclec clone 2100755, Agkisasin-b, Snake venom serine protease Da-36, Agglucetin-alpha 1 subunit precursor, Agkisacutacin B-chain, Metalloproteinase, Zinc metalloproteinase/disintegrin, Snaclec agkisacutacin subunit A, Agkihagin, Thrombin-like protein DAV-PA precursor, Phospholipase B,Antithrombin 1 A chain,Glutaminyl-peptide cyclotransferase, ACF 1/2 B-chain [88]
*Echis carinatus*	1	1.49	Saudi Arabia	Ischemic	Hemiplegia, aphasia	Zinc metalloproteinasedisintegrin-like EoVMP2, Haemorrhagic metalloproteinase, Zinc metalloproteinasedisintegrin-like VLAIP-B, Zinc metalloproteinasedisintegrin-like ACLD, L-amino-acid oxidase, Basic phospholipase A2 Drk-b1, Acidic phospholipase A2 daboiatoxin B chain, Acidic phospholipase A2 EC-I, Serine protease VLSP-1, Phospholipase B, Venom phosphodiesterase 1, Venom serine proteinaselike protein 1, Factor V activator RVV-V alpha, Beta-fibrinogenase, Hyaluronidas, C- type lectin, Disintegrin, Aminopeptidase [113]
*Hypnale* spp	3	4.47	Sri Lanka, India	Ischemic, Hemorrhagic	Alteration of consciousness, hemiparesis	Phospholipase A2, serine proteinases, metalloproteinases, and three-finger toxins [114]
*Protobothrops mucrosquamatus*	1	1.49	Taiwan	Ischemic	Hemiplegia, hypertension, altered consciousness	Metalloproteinases,phospholipases A2, serine proteases, and vasoactive peptides, C-type lectin [115]
*Pseudonaja textilis*	2	2.98	Australia	Ischemic	Dysphagia, respiratory distress, altered, consciousness	Serine proteinase inhibitors, various phospholipase A2s, and pre and postsynaptic neurotoxin [116]
*Trimeresurus*∗∗	2	2.98	India, China	Ischemic	Hemiplegia, aphasia, blurred vision	C-type lectin, Snake venom serine Protease, Phospholipase A2, Glutathione peroxidase, Aminopeptidase, Endonuclease [117]
*Bungarus caeruleus*	1	1.49	India	Ischemic	-Bilateral ptosis, difficulty breathing, headache, nausea	Zinc metalloproteinasedisintegrin BmMP, Acetylcholinesterase, L-amino-acid oxidase, Cysteine-rich secretory protein Bc-CRPb, Muscarinic toxin MTX6, Beta-bungarotoxin A chain precursor [118]
Cobra	1	1.49	India	Ischemic	Breathlessness, hypertension, tachycardia, bilateral ptosis, restricted eye movement	L-amino acid oxidase, serine proteases, metalloproteases, phosphodiesterases, 5′-nucleotidases, cholinesterases, phospholipase B, hyaluronidases, aminopeptidases, phospholipase A_2_ [119]

∗Species identified: *Bothrops marajoensis* (n = 1), *Bothrops atrox* (n = 8).

∗∗Species identified: *Crotalus durissus terrificus* (n = 1), *Crotalus oreganus helleri* (n = 2).

∗∗∗ Species identified: *Trimeresurus gramineus* (n = 1), *Trimeresurus stejnegeri* (n = 1).

**Fig. 2:**
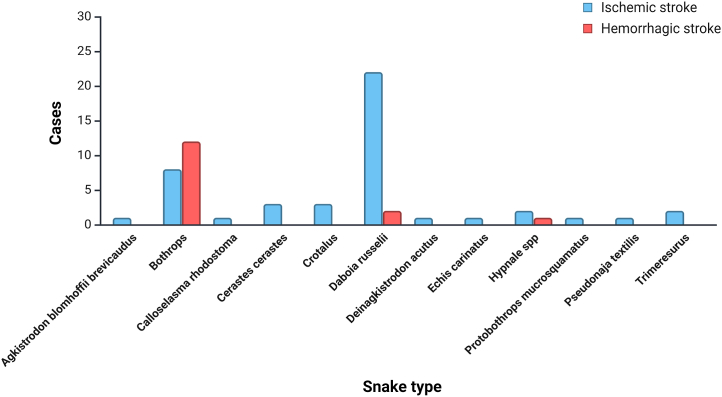
Distribution of Ischemic and Hemorrhagic Stroke Cases by Snake Type.

Regarding the type of stroke, the genus *Bothrops* was responsible for the majority of cases (n = 12) of hemorrhagic stroke; regarding ischemic stroke, *Daboia russelii* was responsible for the majority of cases (n = 24) followed by *Bothrops* (n = 7), *Cerastes cerastes* (n = 5), *Crotalus* (n = 3) and *Hypnale* spp (n = 3) (Fig. 2).

### 3.3 Clinical manifestations and symptom patterns across different snake genus in stroke patients

The analysis of clinical manifestations revealed that the most affected systems were the nervous, cardiovascular, and respiratory systems. Among neurological symptoms, the most frequent was alteration of consciousness, present in 30.49 % of patients, followed by ptosis 21,95 %, aphasia in 15.85 %, and drowsy in 14.63 % of cases. In the cardiovascular system, tachycardia was the most common symptom, affecting 19.51 % of patients, followed by hypertension 18.29 %, and hypotension in 13.41 %. In the respiratory system, the most common manifestation was tachypnea, present in 7.32 % of cases.

**Fig. 3:**
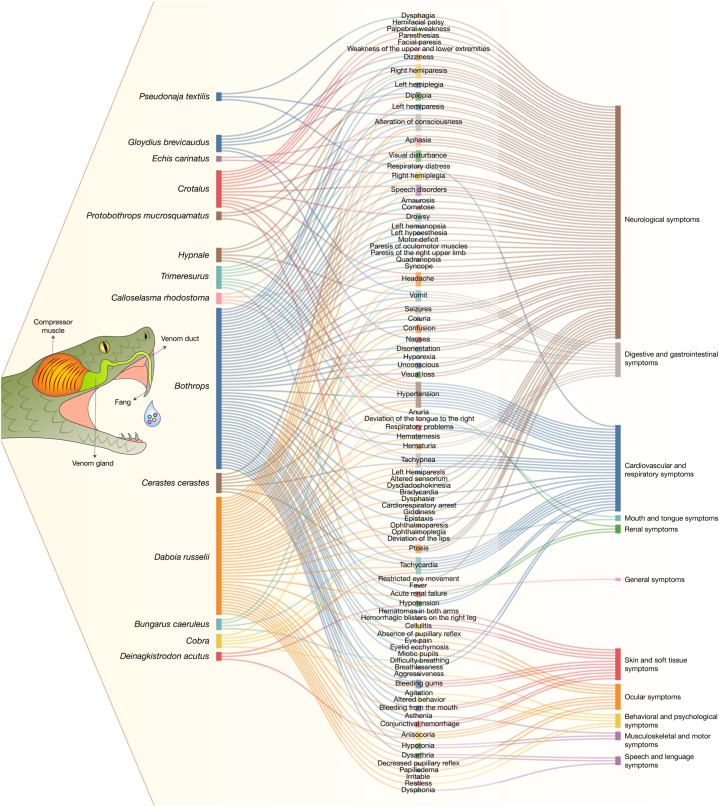
Characterization of Symptoms and Affected Systems by Type of Venomous Snake.

When analyzed by snake genus, *Bothrops* produced the greatest diversity of clinical manifestations, with 56 different symptoms, followed by *Daboia russelii* with 41 symptoms and *Crotalus* with 13 (Fig. 3). The most frequent symptoms across patients were alteration of consciousness (30.40 %), ptosis (21.95 %), tachycardia (19.51 %), hypertension (18.29 %), aphasia (15.85 %), drowsy (14.63 %), bleeding gums (14.64 %), hypotension (13.41 %), and seizures (12.20 %).

In relation to the type of snake responsible, 37.5 % of alteration of consciousness cases were attributed to *Bothrops*, which was also responsible for 28.57 % of hypertension cases, 46.15 % of aphasia cases, 23.52 % of tachycardia cases, and 40 % of seizure cases. Conversely, *Daboia russelii* was responsible for 83.33 % of ptosis cases, 58.33 % of drowsiness cases, 46.15 % of left hemiparesis cases, and 33.33 % of speech disorder cases. In terms of bleeding gums, 85.71 % of cases were linked to *Daboia russelii*.

For other snakes, *Pseudonaja textilis* caused dysphagia and *Trimeresurus stejnegeri* tongue deviation. Snakes from the genus *Crotalus* were the only ones to cause palpebral weakness, paresthesias, and facial paresis (Fig. 3).

### 3.4 Comprehensive imaging findings in ischemic and hemorrhagic strokes following snakebite envenomation

Imaging studies conducted on patients included computed tomography (CT), magnetic resonance imaging (MRI), and multimodal magnetic resonance angiography which were used to diagnose and localize ischemic and hemorrhagic strokes following snakebite envenomation.

In cases of ischemic stroke, CT scans identified infarcts in various brain regions, including the right frontal, parietal, occipital lobes, right basal ganglia, right thalamus, and the internal capsule. Additional findings included infarcts in the right lateral periventricular anterior horn and left frontal, parietal, and occipital lobes. Other common findings were infarcts in the head of the left caudate nucleus, a midline shift, acute ischemic infarcts in the left middle cerebral artery territory, bilateral infarctions of the cerebellum, obstructive hydrocephalus, and bilateral posterior circulation infarcts without hemorrhage. Some scans also revealed infarcts in the internal capsule, capsuloganglionic area, lentiform nucleus, medulla oblongata, and pons with mass effect [30,34,36,37,39,43,48,49,[53], [54], [55], [56], [57], [58], [59], [60],^63^,^68^]. Multimodal magnetic resonance angiography revealed multifocal segmental cerebral artery vasoconstriction, predominantly affecting both the middle cerebral arteries and the P1-segment of the posterior cerebral artery vasoconstriction in the bilateral middle and posterior cerebral arteries with no systemic bleeding segmental areas of luminal narrowing with skip areas, giving a beaded appearance in proximal segments of the bilateral anterior, middle and posterior cerebral arteries [83].

MRI provided more detailed findings, showing infarcts in the right occipital, frontal, temporal, and parietal lobes, as well as in the right sylvian and parasylvian regions, with small infarcts in the right middle cerebral artery territory. Other findings included ischemia in the right insula and right centrum semiovale, as well as infarcts in the left occipital, temporal, and parietal lobes. Additionally, infarctions were detected in the left pontine region, extending to the bilateral tegmentum of the pons, the precentral and postcentral gyrus, and the hemipons. MRI also identified hypo-intensities in both lobes of the cerebellum, bilateral infarcts of the thalamus, a thrombus in the basilar artery, ischemia in the pons, and multiple supra- and infratentorial infarcts affecting the white matter, deep gray matter, nuclei, and cerebellar hemispheres [[32], [33], [34], [35],^38^,[40], [41], [42], [43], [44], [45], [46], [47],[50], [51], [52],^61^,^62^,[64], [65], [66], [67]].

For hemorrhagic strokes, CT scans revealed hemorrhagic lesions in the right parietal, frontal, and temporal lobes, as well as flooding of the right ventricular system and intraparenchymal hemorrhage in the right frontal region. Other findings included hypodense lesions in the right frontal lobe, hematomas in the left temporo-parietal lobe, and the left frontal lobe. Hyperdense areas were found in the posterior horn of the left lateral ventricle, with dilation of the homolateral temporal horn, and evidence of subarachnoid hemorrhage. Other findings included hemorrhages in the cerebellum, angioedema of the bilateral occipital lobes, hemorrhages in the cerebellar tonsil and internal capsule, as well as signs of cerebral edema [60,[69], [70], [71], [72], [73], [74], [75], [76], [77], [78], [79], [80], [81]]. MRI also revealed bilateral and symmetrical periventricular white matter hyperintensities extending to the subcortical white matter [78].

## 4 Discussion

Snakebites are a significant public health issue in many regions, particularly in tropical and subtropical areas where venomous species are prevalent. The clinical presentation of snakebite envenomation varies widely depending on the species involved, the amount of venom injected, and the victim's physiological response. One of the hallmark features of envenomation is swelling, which often triggers a cascade of local and systemic symptoms. Swelling caused by snakebites leads to a range of clinical manifestations, with the most common including localized edema at the bite site, intense pain, ecchymosis, hematoma, blisters, active bleeding, necrosis of the affected limb segment, visual disturbances, dizziness, fainting, nausea, drowsiness, and bleeding from the oral mucosa or digestive tract, among other symptoms ([85]; [14]). Snake venom is a complex mixture of toxins that can have widespread systemic effects, leading to severe complications including neurological damage [86]. Hemotoxins primarily target blood and vascular structures, while neurotoxins disrupt the nervous system, and cytotoxins are responsible for the destruction of cells at the bite site and beyond [13,87]. These toxins can induce significant neurological complications, such as cerebral edema, hemorrhagic stroke, and ischemic stroke, all of which are serious, potentially life-threatening conditions [92]. The exact mechanism by which these cerebrovascular events occur is linked to neurotoxic and hemotoxic enzymes present in the venom, which can lead to disruptions in the coagulation pathway, vascular integrity, and overall cerebral blood flow [13,87].

The frequency of cerebrovascular accidents caused by snake envenomation remains largely unknown. In Ecuador, for example, a study of 309 patients bitten by Bothrops snakes found that 2.6 % developed cerebrovascular events [13]. Another Ecuadorian study involving 294 snakebite cases reported that 5.1 % of patients developed intracranial hemorrhages, although the diagnoses were based solely on clinical observations rather than confirmed imaging [89]. For their part, Al-Sadawi et al. reported in their study that out of 83 cases, 77.1 % had ischemic stroke, 20.5 % had intracranial hemorrhage, and both infarction and hemorrhage were present in 2.4 % [90]. These findings highlight the importance of further research to better understand the prevalence and mechanisms behind snakebite-induced strokes.

In the reviewed cases, all strokes occurred due to bites from front-fanged snakes. The majority were from the *Viperidae* family, with one case caused by a snake from the *Elapidae* family (Table 3).

**Table 3:** Snake venom toxins involved in stroke and their mechanisms of action.

Family	Snake Species	Key Toxins	Mechanism of Action
Viperidae	*Agkistrodon blomhoffii brevicaudus*	Arginine ester hydrolase, lupus anticoagulant-like proteins, hemorrhagins	Arginine ester hydrolase leads to hypercoagulability; hemorrhagins cause vascular damage, increasing the risk of hemorrhagic stroke. Lupus anticoagulant-like proteins interfere with coagulation pathways.
	*Bothrops*	Aspercitin, hemorrhagins, metalloproteases, serine proteases, phospholipases A_2_ and L-amino acid oxidase	Metalloproteinases cause tissue destruction and inflammation; hemorrhagins contribute to vascular leakage, while aspercitin induces platelet aggregation, serine proteases promote blood clotting, phospholipase A_2_ induce myotoxic, neurotoxic, hemolytic, edematogenic, cytotoxic and proinflammatory effects L-amino acid oxidase has cytotoxic and myotoxic effects and can cause hemolysis.
	*Calloselasma rhodostoma*	Metalloproteinase, C-type lectin, serine protease, phospholipase A2, L-amino acid oxidase	Phospholipase A2 disrupts cell membranes leading to inflammation; metalloproteinases cause tissue necrosis, and serine protease promotes coagulation, which can lead to ischemic stroke.
	*Cerastes cerastes*	Metalloproteinases, hemotoxins, phospholipase A2	Hemotoxins damage blood vessels and disrupt normal blood clotting; phospholipase A2 increases cell damage and tissue inflammation, which can result in ischemic stroke.
	*Crotalus*	Arginine ester hydrolase, thrombin-like enzymes	Thrombin-like enzymes induced consumption coagulopathy; arginine ester hydrolase increases clot formation, both raising the risk of ischemic strokes.
	*Daboia russelii*	Proteases	Proteases damage vascular endothelium, contributing to coagulopathy, vascular leakage, and possibly hemorrhagic stroke due to vessel damage.
	*Deinagkistrodon acutus*	Metalloproteinases, serine proteases, C-type lectin proteins, phospholipase A2	These toxins promote blood clotting, disrupt cell membranes, and lead to tissue destruction and inflammation, potentially contributing to both ischemic and hemorrhagic stroke. C-type lectin proteins can
	*Echis carinatus*	Zinc metalloprotein, thrombin-like enzymes	Thrombin-like enzymes accelerate clot formation, while zinc metalloprotein disrupts tissues and blood vessel integrity, potentially leading to stroke.
	*Hypnale* spp	Metalloproteinase, C-type lectin, serine protease, phospholipase A2, L-amino acid oxidase	Similar to other Viperidae, these toxins cause cell membrane damage, coagulation abnormalities, and blood vessel damage, potentially leading to ischemic stroke.
	*Protobothrops mucrosquamatus*	Metalloproteinase, C-type lectin, serine protease, phospholipase A2	Causes tissue necrosis, inflammation, and clotting abnormalities; similar to other viper toxins, they can trigger both ischemic and hemorrhagic strokes.
	*Trimeresurus*	Metalloproteinases, thrombin-like enzymes, α- and β-fibrinogenases	Thrombin-like enzymes promote coagulation, while metalloproteinases and fibrinogenases disrupt the blood clotting cascade, raising the risk of clot formation or bleeding. α- And β-fibrinogenases have the ability to cleave the polypeptide chains of fibrinogen
Elapidae	*Pseudonaja textilis*	Prothrombinase	Prothrombinase significantly enhances clot formation, raising the risk of ischemic strokes.

Table built from: [13,[91], [92], [93], [94], [95], [96]].

These two families are known to contain the most venomous species globally [97]. Notably, no cases involving the *Atractaspididae* subfamily or rear-fanged snakes from “non-front-fanged colubroid snakes” were identified. This may be attributed to the small volume of venom these snakes inject and their inability to deeply penetrate tissue, making serious complications from rear-fanged snake bites relatively rare [92].

The mechanisms through which snake venom can lead to cerebrovascular events are varied and complex. Ischemic stroke is often linked to the procoagulant effects of certain venom components, such as serine proteinases, metalloproteinases, arginine, esterase, and hydrolase, which promote the formation of microthrombi that occlude small or large blood vessels, causing cerebral infarction [52,55]. Hemorrhagins, on the other hand, can induce vasospasms, reducing or entirely preventing proper blood flow. Additionally, direct venom-induced damage to vascular endothelial cells may result in vasculitis, leading to thrombosis [52,55]. Hypotension may develop from bradykinin-enhancing peptides, natriuretic peptides, phospholipase A2, proteases, vascular endothelial growth factors, and three-finger toxins. Other factors contributing to the development of hypotension include hypovolemia due to increased vascular permeability, soft tissue fluid loss, or myocardial depression [98]. The cardiotoxic effects of snake venom can also lead to cardiac thromboembolism, further increasing the risk of stroke [52].

In cases of hemorrhagic stroke, the proteolytic activity of snake venom results in direct damage to the vascular walls, leading to bleeding [13,77]. Metalloproteinase PI, a component of venom, exhibits fibrinolytic and thrombolytic activity, digesting extracellular matrix components and inducing hemorrhage. Additionally, snake venom can cause thrombocytopenia and prolongation of prothrombin and partial thromboplastin times, further promoting bleeding and predisposing patients to hemorrhagic stroke [13,77].

Our review found that the highest number of snakebite-induced stroke cases were reported in Asian countries, particularly India, Brazil, and Sri Lanka. Asia has the highest incidence of snakebites worldwide, with South Asia reporting approximately 121,000 envenomations annually and Southeast Asia 111,000 [5]. In India, an estimated 81,000 snakebites occur each year, followed by 30,000 in Brazil and 33,000 in Sri Lanka [5]. Furthermore, our findings reveal that males are disproportionately affected by snakebite-induced strokes, likely because these demographic experiences the majority of bites. Studies indicate that 69.55 % of cases in India, 88 % in Brazil, and 58.9 % globally occur in men [3,99,100].

Certain risk factors, such as engaging in outdoor activities, significantly increase the likelihood of snakebites and their complications [101,102]. Around 69 % of cases were reported in individuals involved in outdoor work, particularly farming, where 34.5 % of bites occurred [101,102]. Other contributing factors include a lack of preventive measures, with 82.7 % of cases involving individuals who were not wearing boots and 96.5 % in those who did not wear gloves [101,102]. Additionally, environmental factors play a role, with 96 % of cases occurring during the dry season, as opposed to only 4 % in the rainy season [102].

It is crucial to emphasize that the time between a snakebite and the onset of neurological symptoms can vary greatly, ranging from minutes to several hours or even days. In some instances, neurological symptoms have been reported as late as six days post-bite [31,35,46,47,51,65]. As such, patients who have suffered snakebites should remain under prolonged medical supervision, even if they have received antivenom and appear clinically stable. Continuous monitoring is essential to detect early warning signs that could indicate the development of a cerebrovascular event.

Regarding management, guidelines recommend the use of antivenom. However, it should only be administered to patients when the potential benefits are judged to outweigh the associated risks [14]. Treatment with antivenom is recommended if and when a patient with a confirmed or suspected snakebite develops one or more of the following signs: systemic envenomation (hemostatic abnormalities, neurotoxic signs, cardiovascular abnormalities, acute kidney injury, and supporting laboratory evidence of systemic envenoming) [14]. In cases of systemic envenoming, the following should be observed: local swelling involving more than half of the bitten limb, swelling after bites on the digits, rapid extension of swelling, and development of an enlarged tender lymph node draining the bitten limb) [14]. The poison neutralizes the freely circulating venom, but this process takes time; many patients may require life support systems, such as shock treatment, assisted ventilation, and renal dialysis, until severely damaged organs and tissues have had time to recover [14]. The guidelines also recommend using atropine in cases of symptomatic bradycardia, neostigmine in cases of severe neuromuscular blockade prior to the use of mechanical ventilation, and always in conjunction with the application of specific antivenom [85]. Additionally, administration of tetanus toxoid and the use of analgesics such as paracetamol or tramadol are recommended [85].

Despite the existing guidelines for snakebite poisoning management, there is a clear need for a specific protocol or guide addressing stroke management related to snakebites. Current guidelines lack an algorithm tailored to this condition. This gap is evident in the reviewed articles, where treatments largely focus on administering antivenom, tetanus toxoid, atropine, and neostigmine, alongside supportive measures such as mechanical ventilation and dialysis for renal failure. However, these approaches do not provide detailed guidance for the specific management of stroke in this context.

Given the significant and often prolonged impact of snakebites, it is critical for public health systems to ensure comprehensive care and prevention strategies. Patients must be closely monitored after snakebites, even if they appear clinically stable and have received treatment, to prevent delayed-onset cerebrovascular complications. The economic and health consequences of long-term disabilities due to snakebites, particularly strokes, underscore the urgent need for enhanced global awareness, improved treatment protocols, and widespread availability of antivenom in affected regions.

## 5 Limitations

This study has several limitations. First, as it was based on case reports and case series, it is not possible to establish a definitive causal relationship between snakebite envenomation and stroke. Furthermore, the study design limits the ability to identify specific risk factors that may predispose patients to develop stroke following a snakebite or factors that contribute to a fatal outcome. Additionally, several articles did not specify the genus of the snake responsible, raising the possibility that other genera capable of causing stroke may not have been included in this review. In cases where the genus or species of the snake was reported, many studies did not specify the methods used for its identification. In other instances, the identification was based on descriptions by witnesses of the characteristics of the snake that bit the patient, or through the use of photographs of snakes in the region, which compromises the accuracy of the identified species. In some cases, the identification was carried out by a herpetologist, or in other cases, the snake's corpse was taken to the hospital, which are more reliable methods of identification. The reliance on case reports also prevents the establishment of standardized guidelines for the optimal management of patients with stroke resulting from snakebites. Finally, restricting the selection of articles to those published in English and Spanish may have led to the exclusion of relevant studies published in other languages, potentially limiting the comprehensiveness of the review.

## 6 Conclusions

The development of protocols and guidelines for the management of stroke following snakebites is crucial, especially as human activities increasingly encroach upon natural snake habitats due to agricultural expansion and urbanization. Further research is needed to identify which snake species pose the highest risk of causing stroke and to analyze the specific toxins within their venom to better understand the underlying pathophysiological mechanisms. Additionally, understanding the geographic distribution of venomous snakes is essential, particularly in situations where the snake species cannot be identified, allowing healthcare providers to make informed assumptions about the potential culprit. These efforts are vital for improving patient outcomes and reducing the morbidity and mortality associated with snakebite-induced cerebrovascular events.

## CRediT authorship contribution statement

**Jorge Vasconez-Gonzalez:** Writing – review & editing, Writing – original draft, Investigation, Formal analysis, Data curation, Conceptualization. **Karen Delgado-Moreira:** Formal analysis, Data curation. **Juan S. Izquierdo-Condoy:** Investigation, Formal analysis, Data curation. **María de Lourdes Noboa-Lasso:** Data curation. **Esteban Gamez-Rivera:** Supervision, Data curation. **María Belén Lopez-Molina:** Investigation. **Andrés López-Cortés:** Visualization, Software. **Andrea Tello-De-la-Torre:** Visualization, Validation, Software. **Alejandra Torres Cerda:** Data curation. **Daniela Silva Martinod:** Data curation. **Esteban Ortiz-Prado:** Writing – review & editing, Writing – original draft, Conceptualization.

## Ethics approval and consent to participate

Not applicable.

## Consent for publication

Not applicable.

## Availability of data and materials

Not applicable.

## Funding

Universidad de las Americas.

## Declaration of competing interest

The authors declare that they have no known competing financial interests or personal relationships that could have appeared to influence the work reported in this paper.
