# Supplementary material for: Cerebrovascular events induced by venomous snake bites: A systematic review
Source: Heliyon. 2025 Feb 19;11(4):e42779. doi: 10.1016/j.heliyon.2025.e42779 (PMC11904508; doi:10.1016/j.heliyon.2025.e42779)
Supplement: Multimedia component 1 [file mmc1.docx]

**Supplementary material**

**Table S1:** JBI Critical Appraisal Checklist for Case Reports

| **Study** | **1** | **2** | **3** | **4** | **5** | **6** | **7** | **8** | **Score** |
| --- | --- | --- | --- | --- | --- | --- | --- | --- | --- |
| Bashir & Jinkins 1985 (Bashir and Jinkins, 1985) | YES | YES | YES | YES | YES | YES | YES | YES | 8 |
| Lee et al. 2001 (Lee et al., 2001) | YES | YES | YES | YES | YES | YES | YES | YES | 8 |
| Numeric et al 2002 (Numeric et al., 2002) | YES | YES | YES | YES | YES | YES | YES | YES | 8 |
| Thomas et al 2006 (Thomas et al., 2006) | YES | YES | YES | YES | YES | YES | YES | YES | 8 |
| Santos-Soares et al. 2007 (Santos-Soares et al., 2007) | YES | NO | NO | YES | NO | YES | YES | YES | 5 |
| Mugundhan et al. 2008 (Mugundhan et al., 2008) | YES | YES | YES | YES | NO | YES | YES | YES | 7 |
| Narang et al 2009 (Narang et al., 2009) | YES | YES | YES | YES | YES | YES | YES | YES | 8 |
| Machado et al. 2010 (Machado et al., 2010) | YES | YES | YES | YES | YES | YES | YES | YES | 8 |
| Mechán Méndez et al. 2010 (Mechán Méndez et al., 2010) | YES | YES | YES | YES | YES | YES | YES | YES | 8 |
| Gouda et al. 2011 (Gouda et al., 2011) | YES | YES | YES | YES | YES | YES | YES | YES | 8 |
| Deepu et al. 2011 (Deepu et al., 2011) | YES | YES | YES | YES | YES | YES | YES | YES | 8 |
| Ittyachen& Mohan 2012 (Ittyachen and Mohan B., 2012) | YES | YES | YES | YES | YES | YES | YES | YES | 8 |
| Chani et al. 2010 (Chani et al., 2012) | YES | YES | YES | YES | YES | YES | YES | YES | 8 |
| Bhatt et al 2013 (Bhatt et al., 2013) | YES | YES | YES | YES | YES | YES | YES | YES | 8 |
| Vale et al. 2013 (Vale et al., 2013) | YES | YES | YES | YES | YES | YES | YES | YES | 8 |
| Aissaoui et al. 2013 (Aissaoui et al., 2013) | YES | YES | YES | YES | YES | YES | YES | YES | 8 |
| Subasinghe et al 2014. (Subasinghe et al., 2014) | YES | YES | YES | YES | NO | YES | YES | YES | 7 |
| Mahale et al. 2014 (Mahale et al., 2014) | YES | YES | YES | YES | NO | YES | YES | YES | 7 |
| Bush et al. 2014 (Bush et al., 2014) | YES | YES | YES | YES | YES | YES | YES | YES | 8 |
| Gopalan et al. 2014 (Gopalan et al., 2014) | YES | YES | YES | YES | YES | YES | YES | YES | 8 |
| Paul et al. 2014 (Paul et al., 2014) | YES | YES | YES | YES | YES | YES | YES | YES | 8 |
| Rebahi et al. 2014 (Rebahi et al., 2014) | YES | YES | YES | YES | YES | YES | YES | YES | 8 |
| Kumar et al 2015 (Kumar et al., 2015) | YES | YES | YES | YES | YES | YES | YES | YES | 8 |
| Pardal et al. 2015 (Pardal et al., 2015) | YES | YES | YES | YES | YES | YES | YES | YES | 8 |
| Ghezala&Snouda 2015 (Ghezala and Snouda, 2015) | YES | YES | YES | YES | YES | YES | YES | YES | 8 |
| Prabhakar et al. 2016 (Prabhakar et al., 2016) | YES | YES | YES | YES | YES | YES | YES | YES | 8 |
| Cañas 2016 (Cañas, 2016) | YES | YES | YES | NO | YES | YE | YES | YES | 7 |
| Bhojaraja et al. 2016 (Bhojaraja et al., 2016) | YES | YES | YES | YES | YES | YES | YES | YES | 8 |
| Silveira et etl 2016 (Silveira et al., 2016) | YES | YES | YES | YES | NO | YES | YES | YES | 7 |
| Zhang et al 2017(Zhang et al., 2018) | YES | YES | YES | YES | YES | YES | YES | YES | 8 |
| Delgado et al. 2017 (Delgado et al., 2017) | YES | YES | YES | YES | YES | YES | YES | YES | 8 |
| Rathnayaka & Ranathunga 2017 (Rathnayaka and Ranathunga, 2017) | YES | YES | YES | YES | YES | YES | YES | YES | 8 |
| Pothukuchi et al. 2018 (Pothukuchi et al., 2018) | YES | YES | YES | YES | YES | YES | YES | YES | 8 |
| Sahoo &Sriramka 2018 (Sahoo and Sriramka, 2018) | YES | YES | YES | YES | YES | YES | YES | YES | 8 |
| Sahoo et la. 2018 (Sahoo et al., 2018) | YES | YES | YES | YES | YES | YES | YES | YES | 8 |
| Zeng et al. 2019 (Zeng et al., 2019) | YES | YES | YES | YES | YES | YES | YES | YES | 8 |
| Lahiri et al. 2019 (Lahiri et al., 2019) | YES | YES | YES | YES | YES | YES | YES | YES | 8 |
| Pérez-Gómez et al. 2019 (Pérez-Gómez et al., 2019) | YES | YES | YES | YES | YES | YES | YES | YES | 8 |
| Smith and Brown et al. 2019 (Smith and Brown, 2019) | YES | YES | YES | YES | YES | YES | YES | YES | 8 |
| Sachett et al 2020 (Sachett et al., 2020). | YES | YES | YES | YES | YES | YES | YES | YES | 8 |
| Yalcouyé et al 2021 (Yalcouyé et al., 2021) | YES | YES | YES | YES | YES | YES | YES | YES | 8 |
| Ansoumane Hawa et al. 2022 (Ansoumane Hawa et al., 2022) | NO | YES | YES | YES | NO | YES | YES | YES | 6 |
| Martinez-Villota et al. 2022 (Martínez-Villota et al., 2022) | YES | YES | YES | YES | YES | YES | YES | YES | 8 |
| Namal Rathnayaka et al. 2022 (Namal Rathnayaka et al., 2022) | YES | YES | YES | YES | YES | YES | YES | YES | 8 |
| Pinzon et al. 2022 (Pinzon et al., 2022) | YES | YES | YES | YES | YES | YES | YES | YES | 8 |
| Ghosh et al. 2022 (Ghosh et al., 2022) | YES | YES | YES | YES | YES | YES | YES | YES | 8 |
| Ouedraogo et al. 2022 (OUEDRAOGO et al., 2022) | YES | YES | YES | YES | YES | YES | YES | YES | 8 |
| Assamadi et al. 2022 (Assamadi et al., 2022) | YES | YES | YES | YES | YES | YES | YES | YES | 8 |
| Senthilkumaran et al. 2023 (Senthilkumaran et al., 2023) | YES | YES | YES | YES | YES | YES | YES | YES | 8 |
| Bente et al. 2024 (Bentes et al., 2024) | YES | YES | YES | YES | YES | YES | YES | YES | 8 |
| Srinath et al. 2024 (Srinath et al., 2024) | YES | YES | YES | YES | YES | YES | YES | YES | 8 |
| Sun et al. 2024 (Sun et al., 2024) | YES | YES | YES | YES | YES | YES | YES | YES | 8 |
| Nascimento et al. 2024 (Nascimento et al., 2024) | YES | YES | YES | YES | YES | YES | YES | YES | 8 |
| Ladgani et al. 2024 (Lagdani et al., 2024) | YES | YES | YES | YES | YES | YES | YES | YES | 8 |
| Senthilkumaran et al. 2024 (Senthilkumaran et al., 2024) | YES | YES | YES | YES | YES | YES | YES | YES | 8 |

1. Were patient demographics clearly described?

2. Was the patient's history clearly described and presented as a timeline?

3. Was the patient's current clinical condition clearly described at the time of presentation?

4. Were diagnostic tests or evaluation methods and results clearly described?

5. Were interventions or treatment procedures clearly described?

6. Was the post-intervention clinical condition clearly described?

7. Were adverse (harm) or unforeseen events identified and described?

8. Does the case report provide lessons to take away?

Quality scores were categorized into three groups: Low: 1-4, Moderate: 5-7, and High: >8

**Table S2:** JBI Critical Appraisal Checklist for Case Series

| **Study** | **1** | **2** | **3** | **4** | **5** | **6** | **7** | **8** | **9** | **10** | **Score** |
| --- | --- | --- | --- | --- | --- | --- | --- | --- | --- | --- | --- |
| Gawarammana et al. 2009 (Gawarammana et al., 2009) | YES | YES | YES | YES | YES | YES | YES | YES | YES | YES | 10 |
| Pothukuchi et al. 2017 (Pothukuchi et al., 2017) | YES | YES | YES | YES | YES | YES | YES | YES | YES | YES | 10 |
| Dabilgou et al. 2021 (Dabilgou et al., 2021) | YES | YES | YES | YES | YES | YES | YES | YES | YES | YES | 10 |
| Sirur et al. 2022 (Sirur et al., 2022) | YES | YES | YES | YES | YES | YES | YES | YES | YES | YES | 10 |

1. Were there clear criteria for inclusion in the case series?

2. Was the condition measured in a standard and reliable way for all participants included in the case series?

3. Were valid methods of condition identification used for all participants included in the case series?

4. Did the case series include consecutive participants?

5. Did the case series have complete inclusion of participants?

6. Were there clear reports on the demographics of the study participants?

7. Was there a clear reporting of the clinical information of the participants?

8. Were outcomes or case follow-up results clearly reported?

9. Was there clear reporting of demographic information from the presenting sites/clinics?

10. Was the statistical analysis appropriate?

Quality scores were categorized into three groups: Low: 1-4, Moderate: 5-7, and High: >8
